# Voluntary assisted dying: estimating life expectancy to determine eligibility

**DOI:** 10.5694/mja2.51789

**Published:** 2022-11-24

**Authors:** Christopher J Ryan, Ben P White, Cameron L Stewart

**Affiliations:** ^1^ St Vincent's Hospital Sydney Sydney NSW; ^2^ University of New South Wales Sydney NSW; ^3^ Australian Centre for Health Law Research Queensland University of Technology Brisbane QLD; ^4^ University of Sydney Sydney NSW

**Keywords:** Legislation, medical; Suicide, assisted

## Open access

Open access publishing facilitated by The University of Sydney, as part of the Wiley ‐ The University of Sydney agreement via the Council of Australian University Librarians.

## Competing interests

No relevant disclosures.

to the editor: When statutes govern clinical activity, doctors need to know exactly what those legislative provisions mean. Nahm and colleagues^1^ address this in their article on eligibility for Australia's voluntary assisted dying (VAD) laws.^1^ However, in our opinion, the authors misinterpreted the relevant provisions, risking reduced access for eligible patients.

Generally, a statutory provision should be given its “ordinary and natural meaning”;^2^ in other words, a plain English interpretation. As the authors note, each of the VAD Acts uses a particular form of words to set eligibility around a terminally ill person's life expectancy. In Victoria, for example, a coordinating medical practitioner must conclude their patient has been “diagnosed with a disease, illness or medical condition that … is expected to cause death within weeks or months, not exceeding 6 months”.^3^

Nothing in that wording refers to a probabilistic estimation of the percentage chance that the patient will die within 6 months nor any estimation of the best‐case scenario, as Nahm and colleagues suggest. If the Victorian Parliament had wanted this type of estimation, wording reflecting it could have been inserted into the *Voluntary Assisted Dying Act 2017* (Vic). Instead, what is needed is that doctors have an expectation, based on the patient's clinical condition, that the illness will result in death within weeks or months, with the proviso that the number of months that the expectation of death will occur within is 6 or fewer.

That clinical judgement is the beginning and end of this criterion. Although that judgement might be informed by knowledge about survival times and even by knowledge about doctors’ accuracy judging survival times, doctors need only certify that they expect that the patient's illness will cause death within 6 months. Nahm and colleagues are wrong to conclude that this wording might mean that people eligible for VAD would be “those with an expected survival time of 2 months”. That is not what the legislation says, and it is a mistake to introduce elements that are not there. Such an interpretation could, in practice, convert the 6 months test to 2 months for some patients, with the risk of narrowing access to VAD for patients the Parliament intended to be eligible.
